# Liver Biomarkers and Lipid Profiles in Mexican and Mexican-American 10- to 14-Year-Old Adolescents at Risk for Type 2 Diabetes

**DOI:** 10.1155/2017/4262536

**Published:** 2017-07-26

**Authors:** Ana Cecilia Fernández-Gaxiola, Roxana Valdés-Ramos, Kimberly G. Fulda, Ana Laura Guadarrama López, Beatriz E. Martínez-Carrillo, Susan F. Franks, Shane Fernando

**Affiliations:** ^1^Facultad de Medicina, Universidad Autónoma del Estado de México, Paseo Tollocan Esq. Jesús Carranza. Col. Moderna de la Cruz, 50180 Toluca, MEX, Mexico; ^2^Department of Family Medicine, Texas Prevention Institute, University of North Texas Health Science Center, 3500 Camp Bowie Blvd., Fort Worth, TX 76107, USA; ^3^Department of Pediatrics, Texas Prevention Institute, University of North Texas Health Science Center, 3500 Camp Bowie Blvd., Fort Worth, TX 76107, USA

## Abstract

Liver enzymes alanine aminotransferase (ALT) and gamma glutamyl transferase (GGT) are markers for type 2 diabetes mellitus (T2DM); alkaline phosphatase is a marker of liver disease. Mexican-American adolescents are disproportionately affected by T2DM, while in Mexico its prevalence is emerging. We assessed liver biomarkers and lipid profiles among Mexican and Mexican-American adolescents 10–14 years old with high/low risk of T2DM through a cross-sectional, descriptive study (Texas *n* = 144; Mexico *n* = 149). We included family medical histories, anthropometry, and blood pressure. Obesity was present in one-third of subjects in both sites. ALT (UL) was higher (*p* < 0.001) in high-risk adolescents (23.5 ± 19.5 versus 17.2 ± 13.4 for males, 19.7 ± 11.6 versus 15.1 ± 5.5 for females), in Toluca and in Texas (26.0 ± 14.7 versus 20.0 ± 13.2 for males, 18.2 ± 13.4 versus 14.6 ± 10.1 for females), as well as GGT (UL) (*p* < 0.001) (18.7 ± 11.1 versus 12.4 ± 2.3 for males, 13.6 ± 5.8 versus 11.5 ± 3.9 for Mexican females; 21.0 ± 6.8 versus 15.4 ± 5.5 for males, 14.3 ± 5.0 versus 13.8 ± 5.3 for females in Texas). We found no differences by sex or BMI. Total cholesterol and HDL were higher among Mexican-Americans (*p* < 0.001). In conclusion, multiple risk factors were present in the sample. We found differences by gender and between high and low risk for T2DM adolescents in all liver enzymes in both sites.

## 1. Introduction

Type 2 diabetes mellitus (T2DM) is a chronic condition that results from a progressive insulin secretory defect related to insulin resistance caused by a combination of genetic and lifestyle factors [1, 2]. While most important lifestyle factors include unhealthy nutrition and physical inactivity that promote overweight and abdominal obesity [1, 3], genetic factors may vary across populations. It is well established that Mexicans have a high genetic predisposition for T2DM [4, 5]; although genes that predispose an individual to T2DM are considered an essential factor in the development of the disease, activation of a genetic predisposition requires the presence of environmental and behavioural factors, particularly those associated with lifestyles [6].

The increasing worldwide prevalence of overweight, obesity, and metabolic syndrome has revealed that liver enzymes have a potential role as determinants of T2DM and other metabolic conditions such as cardiovascular disease, hyperlipidaemia, and atherothrombotic risk profile [7–9]. Measurement of liver enzymes has become accessible and widely used not only in detecting the incidence, development, and prognosis of liver disease with obvious clinical symptoms but also in assessing overall health status and liver metabolic status [7, 10]. Gamma glutamyl transferase (GGT) is predictive of future diabetes [11–14]. Alanine aminotransferase (ALT) increases with insulin resistance, an independent predictor of T2DM [15, 16]. ALT is the most specific marker of this hepatic pathology [17]. Even within the normal range, both ALT and GGT enzymes have been reported to predict incident diabetes, independent of BMI and alcohol intake [11, 17–19]. However, while some studies have demonstrated a stronger association between GGT and diabetes than between ALT and diabetes [14, 20], others have reported the opposite [21]. A third enzyme, alkaline phosphatase (ALP), is not specific of T2DM but is a marker of liver disease, particularly nonalcoholic fatty liver disease (NAFLD) which is associated with insulin resistance [22, 23]. Reference intervals for all three of them are age and sex related.

Dyslipidaemia occurs in two-thirds of diabetic cases and is mainly characterized by hypertriacylglyceridaemia, low HDL cholesterol, and increased low-density lipoprotein concentrations [24]. There is an association of hyperinsulinemia with hypertriacylglyceridaemia, low HDL, and high LDL [14]. Dyslipidaemia is highly prevalent among Mexicans, and the prevalence of insulin resistance associated with hypertriacylglyceridaemia in the Mexican population, from various studies, ranges from 36.4% to 59% [25].

In Mexico, according to the most recent National Health and Nutrition Survey 2012 (ENSANUT 2012), there are 6.5 million people with diabetes (9.2%), with a rise of nearly 25 percent from 2006 to 2012 [26]. There is an increase in the prevalence of T2DM among Mexican adolescents, and among Mexican-American adolescents living in the US, the increase is even higher [27] (i.e., nearly 1% versus 28%, resp.) [26, 28]. The prevalence of T2DM is disproportionally high among Mexican-Americans compared with other Hispanic groups (5% for Puerto Ricans, 3% for Central Americans, 2% for South Americans, and 1% for Cubans) or with non-Hispanic white Americans [29]. When compared by age group, the prevalence of T2DM among 15- to 19-year-olds is twice the prevalence among 10- to 14-year-olds. In Mexico, the State of Mexico is the second state with the highest prevalence of T2DM in males and the tenth state in females [30]. Toluca is the capital of the State of Mexico.

To our knowledge, no previous study has described serum concentrations of liver enzymes in adolescents at risk for T2DM. As risk factors for T2DM are high among Mexicans and Mexican-Americans, we aimed to assess and compare liver biomarkers and lipid profiles among Mexican and Mexican-American adolescents 10–14 years of age, who are at risk of developing T2DM. We hypothesized that biomarkers and lipid profiles would be higher among Mexican-American adolescents as a biological indicator of their higher prevalence of T2DM.

## 2. Materials and Methods

### 2.1. Study Design and Sample

A cross-sectional study of factors associated with a high risk for T2DM in adolescents 10–14 years of age was designed in collaboration with the University of North Texas Health Science Center (UNTHSC) in Fort Worth and the Center for Research and Graduate Studies in Health Sciences at the Universidad Autónoma del Estado de México (UAEM) in Toluca. The sample included 300 couples of healthy adolescents and their father/mother/guardian, equally distributed between Toluca and Texas. In each site, low- and high-risk adolescents were included (see Participants). We used a probabilistic sample with an alpha of 5%, an expected confidence interval of 95%, and a percentage for completion of 66%. Subjects with chronic diseases such as cystic fibrosis, genetic syndromes, hypo- or hyperthyroidism, and adrenal disease; those who self-reported use of corticosteroids during the previous year of the study, and had a fasting finger-prick glucose ≥ 126 mg/dl or postprandial ≥ 200 mg/dl, which are indicative of diabetes mellitus [31], were excluded.

### 2.2. Participants

We drew our sample from fourth- to ninth-grade students. In Toluca, students enrolled in two public middle schools were invited to participate. In Texas, participants were recruited actively and passively, from paediatrics and family medicine clinics of the North Texas Primary Care Practice-Based Research Network (NorTex) and through community events.

Participants were classified according to their risk status using a noninvasive method, as “high risk” if three or more of the following risk factors for T2DM were present or “low risk” if two or less risk factors for T2DM were present [32]:

1. Family history of T2DM in first and second-degree family members
2. Signs of insulin resistance (i.e., presence of *acanthosis nigricans*)
3. BMI-for-age sex-specific ≥ 95 percentile
4. Family history of hypertension or systolic blood pressure/diastolic blood pressure ≥ 95 percentile
5. Fasting finger prick glucose 100–125 mg/dl (5.6–6.9 mmol/l) [31].

### 2.3. Data Collection

Participants were assigned two appointments in Toluca. During the first appointment, there was a direct interview with the parent (father/mother/guardian) and the adolescent. Each one responded to their corresponding demographic questionnaires, and a screening for finger-prick fasting glucose levels to assess overall risk for diabetes was done to confirm inclusion to the study. Ethnic background was classified depending on parental origin and child's place of birth in both places. During the second appointment, clinical and anthropometric measurements and blood samples were obtained. In Texas, participants were assigned only one appointment to complete all information.

Clinical measurements included blood pressure and the physical examination of the neck to determine the absence/presence of *acanthosis nigricans*. After previous standardization, blood pressure was measured in the dominant arm using the auscultation method and a mercury sphygmomanometer. Hypertension was defined as average systolic blood pressure (SBP) and/or diastolic blood pressure (DBP) greater than or equal to the 95th percentile for sex, age, and height [33]. For the analysis, we used the mean of two systolic/diastolic values taken five minutes apart by the same examiner.

Anthropometric measurements included height, weight, waist, and hip circumferences [34]. We used standard methods by trained personnel. We recorded height by using a portable stadiometer and weight with an electronic scale. We calculated body mass index (BMI-for-age) as weight in kilograms divided by height in squared meters (kg/m^2^). Overweight was defined as a BMI between the 85th and 95th percentile and CDC Standards [35] defined as a BMI equal or above the 95th percentile obesity. We determined central adiposity by measuring waist circumference. Waist circumference risk was defined between the 85th and 90th CDC percentiles for adolescents with the same age and sex [36].

Blood samples were collected to measure lipid profiles and nonfractionated liver enzymes: alkaline phosphatase (ALP), gamma glutamyl transpeptidase (GGT), and alanine aminotransferase (ALT). In Toluca, trained personnel from UAEM obtained blood samples at the school. At UNTHSC, trained personnel obtained blood samples from Quest Diagnostics at the Patient Care Centre. Quest Diagnostics performed all laboratory tests at both sites and we used Quest normative data for adolescents of similar age and sex. All information (questionnaires, blood samples, clinical measurements, and laboratory analyses) were associated with a unique identification number. Only 7% of liver biomarkers and lipids data was missing.

### 2.4. Statistical Analysis

Statistical analyses were performed using the SPSS software 19.0 (Chicago, IL). Data are presented as means and standard deviations (SD) or number and valid percentage for central and spread measurements. *t*-tests and *χ*^2^ tests were used to evaluate differences in adolescents' anthropometric and clinical measurements and biomarkers and lipid profiles. Adolescents were stratified by risk status, sex, and location (Toluca versus Texas) to examine differences between Mexican and Mexican-Americans. Since the proportion of male versus female were different, we compared male versus male and female versus female, allowing for increased internal validity of the study. This may have accounted for the differences in body weight, waist circumference, hypertension, and other possible confounders.

We used three-way ANOVA for testing differences of biological markers and lipid profiles between groups. We also used analysis of covariance to control for BMI and sex as possible confounders between study groups. Statistical significance was accepted at the 0.05 level.

### 2.5. Ethics Approval

The Committee for Ethics and Research at Universidad Autónoma del Estado de México in UAEM, the Institutional Review Board at UNTHSC, and the Toluca school district board of directors approved the protocol. Once informed, a written consent was obtained from both parents or a guardian and assent from the adolescent during an informative meeting with parents and adolescents. All subjects accepted to participate.

## 3. Results

A total of 149 adolescents in Toluca and 144 adolescents in Texas completed the study. All reported confirmed ethnicity. We used reminder telephone calls in Texas and attempts to reschedule missed appointments in schools in Toluca. Missing values generally resulted from inability to obtain blood samples. No adolescent withdrew from the study.

Mean age was similar between Mexican and Mexican-American adolescents (12.02 ± 1.20 years and 11.97 ± 1.52 years for females; 11.83 ± 1.23 years and 11.96 ± 1.37 years for males, resp.). There was a significantly higher proportion of Mexican adolescent females compared with males (61.1% in Toluca and 38.9% in Texas; *p* = 0.032, data not shown). Most females in Toluca were attending middle school while most females in Texas were attending elementary school (*p* < 0.001) (Table 1). The highest prevalence of adolescents classified with high-risk status, both males and females, was found at 12 years of age among the Mexican-Americans and at 14 years of age among the Mexicans (data not shown).

### 3.1. Characteristics of Participants

We found no differences in weight and height in Mexicans adolescents, while height was significantly higher in Texas males compared with females (*p* < 0.001). BMI *z*-score distribution was significantly different in both sites within and between groups (*p* < 0.001, data not shown) (Table 1). In Toluca, waist circumference and percentage body fat were significantly higher (*p* < 0.05) among adolescent females (75.01 ± 10.80 and 25.52 ± 11.74, resp.) compared with adolescent males (71.60 ± 9.49 and 20.93 ± 12.18, resp.). In Texas, percentage of body fat was significantly higher in adolescent females compared with adolescent males (31.41 ± 8.99 versus 27.03 ± 10.85, resp.). Prevalence of obesity was higher than prevalence of overweight in both females and males. In Toluca, obesity was present in 49.45% and 34.48% adolescent females and males. In Texas, obesity was present in 35.71% and 45.94% adolescent females and males. Diastolic blood pressure was different between females and males in Toluca (*p* < 0.001), while systolic blood pressure was different between females and males in Texas (*p* < 0.05).

### 3.2. Risk Factors for T2DM

We assessed five risk factors for T2DM. In Toluca, only the prevalence of known family history of hypertension was different between adolescent females and adolescent males being higher for males (51.7% versus 31.8%, resp.; *p* < 0.05) (Table 2). In Texas, prevalence of known family history of diabetes and presence of *acanthosis nigricans* were significantly higher in adolescent females (82.8% and 55.7%, resp.) than in adolescent males (63.5% and 40.5%, resp.) (*p* < 0.05). Fasting glucose was significantly higher between adolescent females and males in Texas compared with adolescent females and males in Toluca (*p* < 0.05).

### 3.3. Biomarkers

Total cholesterol was significantly different between females and males in Toluca and in Texas (Table 3). No significant differences were found in the prevalence of normal/high biological marker concentrations were observed within each site. Prevalence of high values of high-density lipoproteins (HDL) was higher in Toluca, both in adolescent females and males (72.9% versus 66.0%, resp.) (Table 3).

Significant differences in ALP and GGT serum concentrations were found by site, by sex, and by risk status (*p* < 0.001) (Table 4). Significant differences in ALT serum concentrations were found by sex (*p* < 0.05) and by risk status (*p* < 0.001). Triacylglycerides were significantly higher in adolescents classified as high risk in both locations (*p* < 0.001). ALT was significantly higher in high-risk adolescent males (*p* < 0.001).

The ANOVA tests showed significant differences for ALP and GGT by sex and risk status together and for triacylglycerides by location and risk status together (*p* < 0.05) (Table 4). The analysis of covariance showed no differences when controlling for sex and for BMI with the exception of the concentrations of LDL which was significant by risk status when controlling for BMI (*p* = 0.009) (data not shown).

## 4. Discussion

We aimed to compare two different populations within same age range, based on genetic, behavioural, and cultural similarities. We found a high presence of multiple risk factors for T2DM both in Toluca and in Texas, both in adolescent females and adolescent males 10–14 years of age. This is consistent with the high prevalence of T2DM in Mexican-American adolescents (i.e., 10% total diabetes and 28% total diabetes that is undiagnosed) [28] and the emerging prevalence of T2DM among Mexican adolescents (0.7% in 2012) too [37]. Our results also showed that high-risk adolescents in Toluca and in Texas (both adolescent females and males) have significantly higher serum concentrations of GGT compared with low-risk adolescents in Toluca and in Texas (both adolescent females and males). The presence of multiple risk factors and the higher concentrations of liver enzymes may contribute to the development of T2DM on many of these adolescents classified with high-risk status later in life.

We expected to observe not only higher ALT and GGT concentrations among high-risk adolescents, which we did, but also higher concentrations among Mexican-American adolescents in Texas according to the high prevalence of diabetes there. However, we did not find the latter. An elevation of ALT can be partly explained by higher BMI, waist circumference, and triacylglycerides and lower HDL [9], which is what the adolescents in Texas in our study showed. Another smaller part can be explained by hepatitis infection, alcohol consumption, and/or high transferrin saturation [9]. No adolescent reported to have alcohol consumption or had any disease, and it is not likely that many adolescents would have had hepatitis soon after we took the blood samples. There is evidence suggesting that an increase in ALT is associated with a higher risk for T2DM; however, the studies were performed in high-risk populations and in adults [9, 14, 17– 19, 21, 37, 38]. It seems possible that puberty along with the rapid growth, hormonal changes, and the physiological insulin resistance characteristic of this period of life will influence liver biomarkers [39].

There is evidence that Mexican-American and Mexican adolescents with newly diagnosed T2DM are more likely to have ALT values above the upper normal limit and have higher risk to develop higher ALT concentrations compared with non-Hispanics (whites and American-Americans). However, mean ALT concentrations in our study were lower compared with the Hispanics' values reported before [40]. Although our adolescents were at risk and not diabetic, their ALT concentrations show evidence that there is a window of opportunity for timely preventive actions.

GGT may be a better marker than ALT for the prognosis of T2DM, even within normal ranges, that has been recently proposed [9, 20]. In our study, we found higher GGT concentrations in high-risk Mexican-American adolescents in Texas compared with high-risk Mexican adolescents in Toluca. To our knowledge, ethnicity do not affect GGT concentrations and there is evidence of the association between high GGT values and impaired glucose tolerance particularly in Mexican population. A longitudinal study by Nannipieri et al. [40] in Mexican adults to test the hypothesis that enzymes conventionally associated with liver dysfunction may predict diabetes found that mild elevations in liver enzymes are associated with features of the metabolic syndrome; however, only raised GGT was an independent predictor of deterioration of glucose tolerance to impaired glucose tolerance or diabetes. Therefore, given our findings, we can think there is an increased risk for T2DM among high-risk Mexican-American adolescents in Texas that is consistent with the high prevalence of T2DM there. Furthermore, the higher levels of GGT observed in males compared with females might be reflected in an increased risk for T2DM among Mexican adolescents in Toluca too.

Similar to high GGT serum concentrations, high ferritin concentrations have been found to be associated with insulin resistance, as serum ferritin is a downstream effector of GGT. A combined determination of GGT and ferritin might lead to better predictions in patients with insulin resistance [41]; nonetheless, we did not include ferritin assessment in the study.

Regarding ALP, there is ALP activity in all tissues, particularly liver, bile duct, kidney, bones, intestinal mucosa, and placenta. In our study, we found serum ALP differences by sex and by risk status. However, serum ALP values were not fractionated, and therefore we cannot assure values reflect true liver ALP. High ALP values in children and adolescents can be reflective of bone growth [42]; both high- and low-risk adolescents should be experiencing bone growth at some degree. Therefore, high-risk adolescents are demonstrating metabolic changes reflected in their liver function that are known to signal the development of T2DM.

Our results highlight the need for further understanding the underpinnings of the onset of risk factors for T2DM among adolescent females and males. A better understanding of the cross-cultural differences in the developmental trajectory of T2DM and the pathophysiology are also needed to confirm and understand the predictive value of liver enzymes in relation to T2DM in adolescents.

We observed significant sex differences in all three liver enzyme serum concentrations both in Toluca and in Texas. After controlling for sex and for BMI, our results were similar; only LDL concentrations were different by risk status when controlling for BMI. Differences between males and females have been previously reported in adolescents and are thought to be caused by hormonal differences [42]. As our study was done with adolescents, it is possible that we had hormonal differences by a sex effect and by a puberty effect, so that controlling for sex accounted for the differences in body fat distribution reflected in the BMI of females. We did ask our participants to compare their development to most girls/boys of similar age and found no differences between Mexican-American and Mexican adolescents, both males and females.

The prevalence of diabetes is extremely high among Mexican-American adolescents in Texas compared with Mexican adolescents in Toluca (28% and 0.7%, resp.). Even when our study was done because of its feasibility in Texas and Toluca, given the obesity epidemic in Mexico and in the US, it seems reasonable that environmental factors are activating the development of T2DM not only among Mexican-American adolescents but also among Mexican adolescents. As this was a cross-sectional study, we are unable to ascertain that adolescents with high concentrations of liver enzymes will develop diabetes nor when they will develop it. However, our results suggest liver metabolic dysfunction or abnormalities at an early age, both in girls and boys, probably characterized by liver fat, early liver insulin resistance, and inflammation.

The high presence of risk factors among Mexican adolescents has not reached public awareness, perhaps because the prevalence of T2DM is still low. Therefore, some of them may develop T2DM or other metabolic condition like cardiovascular disease or hypertension, especially those classified as high-risk individuals, and live with its comorbidities and its consequences due to inadequate prevention efforts.

An important limitation to our study was the difference in recruitment between locations. In Toluca, participants were recruited from public elementary and middle schools, and in Texas, participants were recruited from clinics and community events. Participants in Texas may have been unhealthier since they were recruited from clinics, and parents may have been more likely to contact study personnel if they felt their child was at high risk. Thus, the Texas sample is not representative of the general population. Future studies should try to broaden recruitment to improve comparisons and generalizability of the results.

## 5. Conclusions

A great majority of 10- to 14-year-old adolescent females and males in Toluca and in Texas have multiple risk factors for T2DM that may lead to metabolic complications. Most of them can be prevented and reverted to avoid the onset of T2DM. We found gender differences and differences between high- and low-risk adolescents in GGT, ALP, and ALT concentrations both in Toluca and in Texas.

## Figures and Tables

**Table 1:** Characteristics of the 10- to 14-year-old Mexican and Mexican-American adolescents by sex and location.

Characteristics	Adolescents					
	Toluca (*n* = 149)			Texas (*n* = 144)		
	Females (*n* = 91)	Males (*n* = 58)	*p* ^∗^	Females (*n* = 70)	Males (*n* = 74)	*p* ^∗^
Age, years	12.02 ± 1.20	11.83 ± 1.23	0.431	11.97 ± 1.52	11.96 ± 1.37	0.590
Education						
Elementary school (%)	46.1	58.6	0.000	51.4	48.6	0.047
Middle school (%)	53.8	41.3		48.5	51.3	
Weight (kg)	52.35 ± 13.68	49.72 ± 14.17	0.263	54.31 ± 15.88	59.90 ± 21.21	0.077
Height (m)	149.71 ± 6.51	148.27 ± 8.83	0.256	150.17 ± 7.00	155.61 ± 11.76	0.001
BMI (kg/m^2^)	23.17 ± 5.26	22.24 ± 5.01	0.283	23.86 ± 6.01	24.08 ± 5.83	0.824
BMI-for-age z-score	0.88 ± 1.16	0.92 ± 1.38	0.120	1.08 ± 1.20	1.24 ± 0.98	0.038
BMI-for-age z-score distribution^1^						
Normal weight	−0.16 ± 0.91	−0.17 ± 1.50	0.000	−0.13 ± 1.09	−0.05 ± 0.55	0.000
Overweight	1.24 ± 0.74	1.34 ± 0.36	0.000	1.37 ± 0.55	1.31 ± 0.22	0.000
Obese	1.76 ± 0.39	1.93 ± 0.45	0.000	2.05 ± 0.19	2.04 ± 0.42	0.000
Waist circumference (cm)	75.01 ± 10.80	71.60 ± 9.49	0.051	78.61 ± 12.66	82.57 ± 15.63	0.099
% body fat	25.52 ± 11.74	20.93 ± 12.18	0.022	31.41 ± 8.99	27.03 ± 10.85	0.010
Systolic blood pressure (mmHg)	115.24 ± 12.98	106.26 ± 9.54	0.251	107.53 ± 10.01	114.22 ± 22.05	0.037
Diastolic blood pressure (mmHg)	70.22 ± 11.18	63.67 ± 10.61	0.000	64.52 ± 5.63	65.23 ± 5.93	0.351
Blood pressure classification^2^			0.297			0.824
Normal tension, % (*n*)	82.4 (75)	58.6 (34)		81.4 (57)	49.4 (37)	
Prehypertension, % (*n*)	6.7 (6)	17.2 (10)		7.1 (5)	12.0 (9)	
Stage 1 Hypertension, % (*n*)	6.7 (6)	15.5 (9)		8.5 (6)	26.3 (19)	
Stage 2 Hypertension, % (*n*)	4.0 (4)	8.6 (5)		2.8 (2)	12.0 (9)	
Age at menarche (females only)	11.38 ± 0.88	—	—	11.02 ± 1.03	—	—

Data are presented as mean ± standard deviation value unless otherwise specified. ^1^Normal weight between the 95th and 85th percentile; overweight between the 85th and 95th percentile; obesity > 95th percentile. ^2^Normal tension < 90th percentile; prehypertension between the 90th and 94th percentile; stage 1 hypertension < 95th percentile; stage 2 hypertension < 99th percentile. ^∗^*p* value from overall test of association between Mexicans and Mexican-American adolescents' characteristics (*χ*^2^ test for categorical variables, Student's *t*-test for continuous variables).

**Table 2:** Number of risk factors for T2DM present in 10- to 14-year-old Mexican and Mexican-American adolescents by sex and location.

Risk factors	Adolescents					
	Toluca (*n* = 149)			Texas (*n* = 144)		
	Female (*n* = 91)	Males (*n* = 58)	*p* ^∗^	Females (*n* = 70)	Males (*n* = 74)	*p* ^∗^
Family history of diabetes (%)	61.5 (56)	56.9 (33)	0.347	82.8 (58)	63.5 (47)	**0.007**
Family history of hypertension (%)	31.8 (29)	51.7 (30)	**0.021**	1.4 (1)	2.7 (2)	0.262
BMI-for-age < 95th percentile (%)	42.8 (39)	31.0 (18)	0.134	31.4 (22)	35.1 (26)	0.234
*Acanthosis nigricans*, present (%)	37.4 (34)	32.7 (19)	0.347	55.7 (39)	40.5 (30)	**0.049**
Fasting glucose of 100–120 mg/dl (%)	25.3 (23)	24.1 (14)	0.330	40.0 (28)	51.4 (38)	0.059
Number of T2DM risk factors present						
0	16	7	**0.041**	6	13	0.056
1	23	22	0.270	25	23	0.467
2	13	7	**0.024**	18	16	0.194
3	25	13	**0.037**	16	17	0.691
4	13	7	**0.025**	5	4	0.091
5	1	2	0.368	0	1	0.211

^∗^ *p* value from overall test of association between Mexicans and Mexican-American adolescents' with *χ*^2^ test. Significant values shown in bold.

**Table 3:** Mean ± standard deviation of serum concentrations of biological markers in 10- to 14-year-old Mexican and Mexican-American adolescents by location.

Biological markers	Normal^1^ Mean ± SD (%)	Borderline^2^ Mean ± SD (%)	High^3^ Mean ± SD (%)
*Toluca*			
Total cholesterol (mg/dl)^∗^ Triacilglycerides (mg/dl)	143.40 ± 16.03 (76.7) 89.28 ± 28.61 (76.1)	179.30 ± 7.05 (17.8) 164.64 ± 12.88 (7.4)	229.43 ± 24.19 (5.4) 278.79 ± 64.52 (16.4)
Cholesterol-HDL ratio	3.53 ± 0.64 (91.5)	N/A	5.55 ± 0.73 (8.5)
High-density lipoprotein (HDL) (mg/dl)^∗∗^	46.32 ± 11.27 (72.9)	41 ± 8.31 (8.5)	33.19 ± 5.12 (18.6)^4^
Low-density lipoprotein (LDL) (mg/dl)	78.41 ± 17.59 (88.3)	115.60 ± 5.23 (7.8)	151.80 ± 7.25 (3.9)
Alkaline phosphatase (ALP) (mg/dl)	245.54 ± 106.7 (98.4)	N/A	487.00 ± 7.07 (1.6)
Gamma glutamyl transpeptidase (GGT) (mg/dl)	12.09 ± 3.36 (92.2)	N/A	31.30 ± 8.43 (7.8)
Alanine aminotransferase (ALT) (mg/dl)	16.54 ± 8.28 (95.8)	N/A	62.33 ± 16.58 (4.2)
*Texas*			
Total cholesterol (mg/dl)^∗^ Triacylglycerides (mg/dl)	142.52 ± 16.03 (68.1) 93.83 ± 29.15 (66.7)	180.28 ± 8.40 (27.8) 167.29 ± 15.60 (21.5)	208 ± 9.44 (4.1) 253.76 ± 53.16 (11.8)
Cholesterol-HDL ratio	3.32 ± 0.73 (93.1)	N/A	6.08 ± 1.43 (6.9)
High-density lipoprotein (HDL) (mg/dl)^∗∗^	49.74 ± 9.98 (66.0)	42.89 ± 9.75 (21.5)	35.00 ± 3.40 (11.8)^4^
Low-density lipoprotein (LDL) (mg/dl)	79.13 ± 17.19 (91.0)	116.33 ± 5.31 (8.3)	158.00 ± 0.00 (0.7)
Alkaline phosphatase (ALP) (mg/dl)	225.54 ± 87.82 (100.0)	N/A	0
Gamma glutamyl transpeptidase (GGT) (mg/dl)	14.18 ± 4.24 (89.6)	N/A	27.87 ± 5.73 (10.4)
Alanine aminotransferase (ALT) (mg/dl)	15.80 ± 6.65 (92.4)	N/A	56.64 ± 12.38 (7.6)

^1^Normal values: total cholesterol < 170 mg/dl; LDL < 110 mg/dl; HDL cholesterol ratio < 45 mg/dl; ALP 91–476 UL for males and 104–471 UL for females; GGT 8–32 UL for males and 7–18 UL for females; ALT 8–50 UL for males and 7–33 UL for females. ^2^Borderline values: total cholesterol 170–199 mg/dl; HDL 45–40 mg/dl; LDL 110–129 mg/dl; HDL cholesterol ratio 35–45 mg/dl. No borderline values for ALP, GGT, and ALT available. ^3^High/low values: total cholesterol ≥ 200 mg/dl; HDL ≤ 35 mg/dl; LDL ≥ 130 mg/dl; HDL cholesterol ratio ≤ 35 mg/dl; ALP, GGT, and ALT below or above normal values for males and females. N/A: not applicable. ^∗^*p* < 0.05 between males (*p* = 0.014); ^∗∗^*p* < 0.05 between sites (*p* = 0.012). ^4^Low nonhigh values for high-density lipoproteins.

**Table 4:** Biological markers in 10- to 14-year-old Mexican and Mexican-American adolescents by location, sex, and risk status.

Biomarkers	Toluca (*n* = 149)				Texas (*n* = 144)				Low versus high Risk *p*^∗∗^	Toluca versus Texas *p*^∗∗^	♂ versus ♀ *P*^∗∗^	Risk-location-sex *P*^∗^
	Low risk (*n* = 74)		High risk (*n* = 75)		Low risk (*n* = 99)		High risk (*n* = 45)					
	Male Mean ± SD (*n* = 32)	Female Mean ± SD (*n* = 42)	Male Mean ± SD (*n* = 26)	Female Mean ± SD (*n* = 49)	Male Mean ± SD (*n* = 52)	Female Mean ± SD (*n* = 47)	Male Mean ± SD (*n* = 22)	Female Mean ± SD (*n* = 23)				
Total cholesterol (mg/dl)	125.3 ± 58.9	144.7 ± 48.4	122.6 ± 64.3	135.6 ± 62.2	152.0 ± 24.7	159.4 ± 23.3	158.4 ± 20.1	153.8 ± 29.5	0.621	**0.000**	0.114	0.799
HDL (mg/dl)	46.5 ± 11.1	46.2 ± 10.7	42.1 ± 9.7	39.1 ± 6.2	48.1 ± 13.0	50.7 ± 11.3	41.6 ± 8.7	41.8 ± 10.7	**0.000**	0.128	0.910	0.959
LDL (mg/dl)	82.8 ± 18.8	89.3 ± 24.5	73.6 ± 27.2	85.3 ± 22.8	78.8 ± 19.9	83.0 ± 20.0	90.2 ± 16.2	83.9 ± 24.5	0.927	0.664	0.153	0.159
Triacylglycerides (mg/dl)	96.1 ± 67.8	100.9 ± 58.4	154.9 ± 109.7	169.2 ± 76.8	125.0 ± 59.5	128.2 ± 64.9	132.6 ± 51.9	140.0 ± 79.9	**0.000**	0.896^**&**^	0.411	0.883
Cholesterol/HDL (mg/dl)	3.3 ± 0.6	12.1 ± 54.0	3.7 ± 0.8	4.3 ± 1.8	3.3 ± 0.9	3.3 ± 1.1	3.9 ± 1.0	3.8 ± 0.9	0.561	0.388	0.381	0.442
ALP (mg/dl)	298.8 ± 86.6	268.9 ± 102.2	292.7 ± 110.2	177.5 ± 96.0	259.9 ± 63.8	196.5 ± 94.6	250.8 ± 84.3	183.8 ± 90.1	**0.011**	**0.002**	**0.00** ^**+**^	0.079
GGT (mg/dl)	12.4 ± 2.3	11.5 ± 3.9	18.7 ± 11.1	13.6 ± 5.8	15.4 ± 5.5	13.8 ± 5.3	21.0 ± 6.8	14.3 ± 5.0	**0.000**	**0.006**	**0.00** ^**#**^	0.782
ALT (mg/dl)	17.2 ± 13.4	15.1 ± 5.5	23.5 ± 19.5	19.7 ± 11.6	20.0 ± 13.2	14.6 ± 10.1	26.0 ± 14.7	18.2 ± 13.4	**0.001**	0.605	**0.002**	0.926

^∗^Three-way ANOVA; ^∗∗^Student's *t*-test. Statistically significant by sex and risk status together; ^+^*p* = 0.020; ^#^*p* = 0.018; ^&^statistically significant by location and risk status together, *p* = 0.045. Significant values are shown in bold.
